# Evaluation of tumour marker utilisation and impact of electronic gatekeeping in the province of KwaZulu-Natal, South Africa

**DOI:** 10.4102/ajlm.v12i1.2027

**Published:** 2023-06-30

**Authors:** Immaculate S. Dlamini, Verena Gounden, Nareshni Moodley

**Affiliations:** 1 Department of Chemical Pathology, Faculty of Laboratory Medicine, National Health Laboratory Service, Durban, South Africa; 2 Department of Chemical Pathology, Faculty of Laboratory Medicine, University of KwaZulu-Natal, Durban, South Africa

**Keywords:** tumour marker, demand management, electronic gatekeeping, minimum retesting interval, cost reduction

## Abstract

**Background:**

Inappropriate testing remains a high healthcare cost driver. Tumour marker tests are more expensive than routine chemistry testing. Implementing test demand management systems like electronic gatekeeping (EGK) has reportedly decreased test requests.

**Objective:**

This study aimed to describe the appropriateness of tumour marker tests, carcinoembryonic antigen, alpha foetal protein, prostate-specific antigen, carbohydrate antigen 19-9, cancer antigen 15-3, cancer antigen 125, and human chorionic gonadotropin, and determine the effectiveness of the EGK used in the public health sector in KwaZulu-Natal, South Africa.

**Methods:**

Tumour marker test data for the KwaZulu-Natal province were extracted from the National Health Laboratory Service Central Data Warehouse for 01 January 2017 – 30 June 2017 (pre-EGK) and 01 January 2018 – 30 June 2018 (post-EGK implementation). Questionnaires were sent to the clinicians in the regional hospitals ordering the most tumour marker tests to assess ordering practices. In addition, we assessed monthly rejection reports to determine the effect of the EGK.

**Results:**

The EGK minimally reduced tumour marker requests or associated costs (1.4% average EGK rejection rate). An overall 18% increase in the tumour marker tests occurred in 2018. The data suggest inappropriate tumour marker test utilisation, particularly for screening.

**Conclusion:**

The introduction of EGK as a test demand management had little impact on tumour marker test requests and costs. Continuous education and reiteration of indications for tumour marker test use are required.

**What this study adds:**

This study demonstrates the ineffectiveness of EGK in tumour marker orders, and provides some insight as to why these markers are being ordered, which is important in trying to decrease inappropriate ordering of these tests.

## Introduction

Serum tumour markers are biochemical markers released by tumour cells directly or indirectly as a source or effect of malignant development. Tumour markers are a less invasive tool than a biopsy and are used to increase or decrease the clinical suspicion of a developing new or secondary cancer, detect the recurrence or progression of cancer, monitor response to treatment, and identify a specific therapeutic modality. Ideally, requesting and testing a tumour marker should allow for effective patient management, when appropriately performed, and reduce unnecessary redundant costs.^1^ Therefore, the measurement of non-invasive serum tumour markers has been pursued to expedite early diagnosis and detection of cancer aimed at reducing cancer morbidity and mortality. However, the diagnostic sensitivity and specificity of most currently available serum tumour markers are limited.^2^

The inappropriate use of serum tumour markers has been reported.^3,4,5^ The improved analytical sensitivity and specificity of high-output automated platforms have increased accessibility to the use of tumour markers and increased the use of serum tumour markers.^6^ However, the progression in instrumentation has been incongruent with the adoption of evidence-based guidelines to guide the appropriate use of tumour markers.^7^

The cost of inappropriate testing of tumour markers indirectly affects patient safety, depending on the management strategies initiated based on the results reported.^8^ Increased healthcare costs with decreased healthcare budgets have forced laboratories to develop strategies to reduce and prevent inappropriate testing.^9^

Demand management is one strategy that focuses on ensuring appropriate requests while ensuring quality care to the patient. Laboratories have adopted several strategies to limit test demand, such as requiring requesting physicians to have a predetermined educational level, redesigning test request forms, and physical and electronic-based gatekeeping and reflex testing.^9^

Most serum tumour markers are not recommended as first-line rule-in or rule-out tests for cancer, but rather for detecting tumour recurrence and monitoring treatment. Thus, demand management strategies have been developed.^9,10^ Some strategies have been validated for serum tumour marker testing,^11^ such as the minimum retesting interval (MRI). The MRI strategy stipulates the minimum time before repeating a test based on the test’s properties, such as clinical indication.^12,13^

The South African National Department of Health, in conjunction with the National Health Laboratory Service, which serves the South Africa public health sector facilities, have used the MRI strategy, termed electronic gatekeeping (EGK), to manage test demand since 2017. Electronic gatekeeping was introduced to limit healthcare spending on ‘unnecessary’ laboratory investigations. The criteria for selecting the MRI were based on a combination of literature and consensus agreement between expert clinicians representing the Department of Health in each region, and expert pathologists.

Electronic gatekeeping implementation studies have demonstrated that EGK is an effective cost-saving tool for several laboratory tests. In 2010, Tygerberg Hospital management and the National Health Laboratory Service conducted a pilot project in Cape Town, South Africa, to identify the number of EGK-rejected and EGK-restored (i.e., approved for analysis) tests, the costs saved, and the impact test rejections. The study concluded that the EGK was an effective and sustainable demand management tool. They found that most rejected tests were not restored, revealing the inappropriateness of those test requests. The use of EGK did not appear to negatively impact patient care but was an effective cost-saving tool.^14^

However, an academic hospital in Gauteng province, South Africa, reported that EGK test demand management does not dramatically influence requesting behaviour or save costs. They reported an unchanged monthly percentage of EGK-held tests over a 22-month retrospective study period, suggesting that a solitary demand management strategy is not as effective as anticipated or as demonstrated in other studies.^15^

Both the Cape Town and Gauteng studies only reviewed the effect of routine chemistry testing demand, not tumour marker testing. To date, no study has reviewed the requesting nature of tumour markers in the South African public health sector. In the South African public healthcare sector, all laboratory and other diagnostic costs are borne by the state.

Tumour markers were chosen as they are one of the most highly requested tests in the chemistry laboratory, are more costly to process, and are thus charged at a higher rate than the more routine general chemistry testing. At the time of the study, the National Health Laboratory Service Chemical Pathology laboratory at Laboratory A provided tumour marker testing services for most patients in the public sector covering the entire province of KwaZulu-Natal, except a more northern region, where testing is provided by Laboratory B, a National Health Laboratory Service laboratory, which provides a smaller tumour marker test repertoire. The serum tumour markers that were assessed during this audit were carcinoembryonic antigen, alpha foetal protein (AFP), prostate-specific antigen (PSA), carbohydrate antigen 19-9 (CA 19-9), cancer antigen 15-3 (CA 15-3), cancer antigen 125 (CA 125), and human chorionic gonadotropin (HCG).

Disorders with high AFP include hepatocellular carcinoma, hepatoblastoma, non-seminomatous testicular germ cell tumours of the embryonal carcinomas, cancers of the pancreas, lung, and gastric, and non-malignant processes such as acute viral hepatitis, liver cirrhosis, and obstructive jaundice.^2,16,17^ Carcinoembryonic antigen is a tumour marker for gastrointestinal cancers, but it is also elevated in breast, lung and liver cancers, and non-malignant conditions like heavy smoking, bronchitis, gastritis, duodenal ulcer, liver diseases, pancreatitis, and colorectal polyposis.^16,18^ Human chorionic gonadotropin is produced by embryonal tissue^1^ but is used as a tumour marker in seminomatous and non-seminomatous testicular tumours, ovarian germ cell tumours, the gestational hydatid form mole, choriocarcinoma, and non-testicular teratomas.^19^ Carbohydrate antigen 19-9 is normally synthesised by the pancreas, biliary ductal cells, gastric, colon, and endometrial and salivary epithelia. It is mainly used to prognosticate and monitor response to interventions in patients with pancreatic and gastrointestinal cancer.^1^ Increased CA 125 values most often are associated with epithelial ovarian cancer, although levels can also be increased in other malignancies, such as breast, endometrial, cervix, peritoneal, uterus, lung, pancreas, hepatocellular and non-Hodgkin’s lymphoma and multiple benign disorders, which include pregnancy, endometriosis, uterine fibroids, pancreatitis, normal menstruation, pelvic inflammatory disease, and cirrhosis of the liver.^1^ Cancer antigen 15-3 levels have been reported to be useful to prognosticate in breast cancer patients,^20^ but elevations of CA 15-3 levels are also seen in other malignancies, including pancreatic, lung, ovarian, colon, and liver cancer as well as benign breast and liver conditions.^1^ Prostate-specific antigen aids in the diagnosis, risk assessment, and monitoring of prostate carcinoma, but it is also elevated in non-malignant conditions like acute urinary retention, benign prostatic hyperplasia, prostatitis, and urinary tract infection.^1^

This study aimed to describe the tumour marker requesting practices across different levels of healthcare in the province of KwaZulu-Natal, South Africa, assess the effect of EGK on these requesting practices and, lastly, determine via questionnaire the rationale for tumour marker requesting by the clinicians at the highest ordering facilities.

## Methods

### Ethical considerations

Ethics approval for this study was obtained from University of KwaZulu-Natal Biomedical Research Ethics Committee (number BE035/18). Written informed consent was received from the participating clinicians. Data were collected on a password-protected computer and the primary investigator was the only person with access to it. Patients were identified by hospital numbers and their identities were not revealed. Questionnaires were anonymised and identified by numbers allocated to the specific sites. Survey respondents were assured raw data would remain confidential and would not be shared.

### Data collection

The National Health Laboratory Service Central Data Warehouse reposits all test results generated by National Health Laboratory Service laboratories. We extracted from the National Health Laboratory Service Central Data Warehouse all tumour marker tests performed in public healthcare facilities in KwaZulu-Natal province, South Africa. The data extracted included requests made 6 months pre-EGK (01 January 2017 to 30 June 2017) and 6 months post-EGK implementation (01 January 2018 to 30 June 2018). The data consisted of results from Laboratory A and B National Health Laboratory Service Chemical Pathology laboratory. Laboratory A offers the following tumour marker tests: carcinoembryonic antigen, AFP, PSA, CA 19-9, CA 15-3, CA 125, and HCG, whereas Laboratory B offers all the above tests except CA 19-9. Both laboratories analysed tumour markers on the Siemens Advia Centaur XP (Siemens, Tarrytown, New York, United States). The MRI rules that were in use for EGK per tumour marker test were as follows: HCG: 1 day; AFP: 1 month; PSA: previous result abnormal = 1 month and previous result normal = 1 year; carcinoembryonic antigen: 1 month; CA 125: 1 month; CA 19-9: 1 month; and CA 15-3: 1 month; where: 1 day is 12 h, 1 month is 21 days, and 1 year is 322 days since daily tests are not performed at the same time each day and a repeat visit within a set interval (eg. week or month) may happen before the exact interval has passed. The monthly EGK rejections reports were assessed from 01 January 2018 to 30 June 2018, to determine the number of requests rejected by gatekeeping in the included laboratories.

The top five highest requesting units from all healthcare facilities over the period of the study were identified and chosen as the sites for questionnaire distribution. Written informed consent and the questionnaire (adapted from McGinley and Kilpatrick^21^) were hand delivered and obtained from clinicians from these selected facilities. The questionnaire identified who was ordering tumour markers and why. Responses were collated in Microsoft Excel (Microsoft Corporation, Redmond, Washington, United States) for further evaluation.

### Data analysis

Statistical analyses were performed using Medcalc R version 18.11 (Medcalc Software, Mariakerke, Belgium) and Microsoft Excel 2016 (Microsoft Corporation, Redmond, Washington, United States). Data were assessed for normality using the Shapiro-Wilk test. Normal data were presented as mean ± standard deviation. The monthly average rejection rate was calculated. Costing was done using on the National Health Laboratory Service State Price List 2017.

## Results

A total of 38 615 tumour marker tests for the specified analytes were processed during the 6-month pre-EGK introduction period (01 January 2017 to 30 June 2017), while 45 567 tumour markers requests were processed in the post-EGK implementation period (01 January 2018 to 30 June 2018). In 2018, there was an 18% increase in tumour marker tests processed. The most ordered tumour marker was PSA (41.1% of tested tumour markers in 2017, and 38.4% in 2018), while the least ordered was CA 15-3 (< 3% of tested tumour markers in both 2017 and 2018) (Table 1).

**TABLE 1:** Summary of tumour markers processed in KwaZulu-Natal, South Africa, 01 January 2017 to 30 June 2018.

Analyte	1 January 2017 to 30 June 2017		1 January 2018 to 30 June 2018	
	Absolute number of requests	%	Absolute number of requests	%
Total	38 615	-	45 567	-
PSA	15 870	41.1	17 518	38.4
CEA	5188	13.4	6513	14.3
AFP	5084	13.2	5674	12.4
CA 125	4929	12.8	5854	13.0
CA 19-9	3499	9.0	4801	10.5
BHCG	3088	8.0	4093	9.0
CA 15-3	957	2.5	1114	2.4

PSA, prostate-specific antigen; AFP, alpha foetal protein; CEA, carcinoembryonic antigen; CA 19-9, carbohydrate antigen 19-9; CA 125, cancer antigen 125; CA 15-3, cancer antigen 15-3; BHCG, beta-human chorionic gonadotropin.

The majority of samples for PSA, AFP, carcinoembryonic antigen, CA 19-9, CA 15-3 and CA 125 had normal results (defined as results within the reference interval) in both years of the study period. In contrast, HCG had the most abnormal results (outside of reference interval) for both years in the study period, with 90.1% (2783/3088) abnormal results for 2017, and 92.1% (3771/4092) for 2018.

**FIGURE 1:**
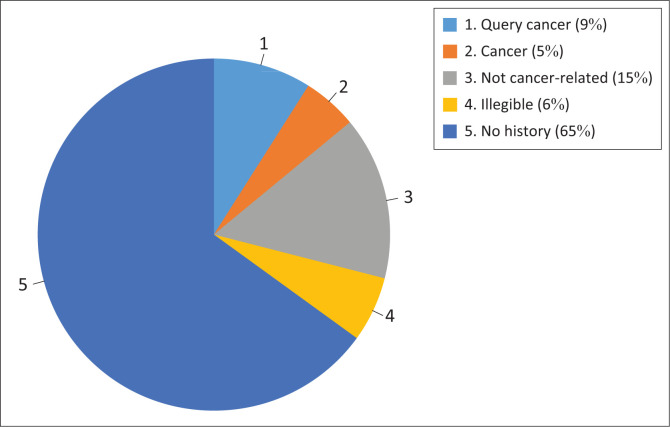
Clinical details for tumour marker test requests retrieved from Laboratory Information System in KwaZulu-Natal, South Africa, 01 January 2018 – 30 June 2018.

Clinical history data provided on the laboratory information system via the Central Data Warehouse demonstrated that there were no clinical histories recorded for most samples (*n* = 21 299, 65%). A minority of samples (*n* = 1535, 5%) had a history of cancer documented on the request forms. Nine percent (*n* = 2927) of requests indicated suspicion of malignancy or screening as request reason (Figure 1).

**FIGURE 2:**
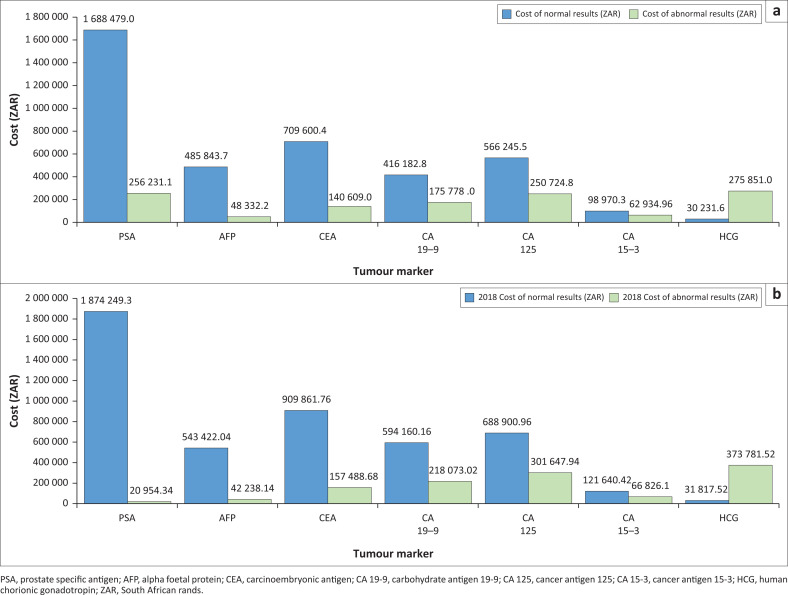
Cost of resulted normal and abnormal tumour markers test requests in (a) 2017 and (b) 2018 in KwaZulu-Natal, South Africa. All cost amounts are shown in South African rand (ZAR).

Based on the National Health Laboratory Service State Price List 2017, the cost of normal results was markedly higher than abnormal results for both study periods. For example, from 01 January 2017 to 30 June 2017, normal results cost 3 995 553.00 South African rand (R) ($218 409.51 United States dollars [USD]), while abnormal results cost R1 210 461.00 ($66 167.61 USD). In the same period in 2018, normal results cost R4 764 052.00 ($260 418.09 USD) and abnormal results cost R1 181 010.00 ($64 557.73) (Figure 2).

Most test requests were received from the outpatient departments or non-oncology clinics of the respective healthcare facilities. However, test requests from the oncology wards and clinics were the lowest. The tertiary academic hospitals made the fewest requests, followed by primary healthcare facilities. Requests from district hospitals increased in 2018 by 58.7% to overtake regional hospitals as the main requestors (Table 2).

**TABLE 2:** Distribution of samples processed per location for tumour marker testing in KwaZulu-Natal, South Africa, 01 January 2017 – 30 June 2018.

Requesting location	Total tumour marker requests†	Total tumour marker requests‡
Surgical wards	2537	2653
Medical wards	3629	7644
Oncology wards/clinics	1753	1522
OPD/clinics	12 885	10 172
Emergency unit/casualty	2517	3548
Other	9097	12 022
Tertiary level healthcare	4916	5141
Regional level healthcare	13 229	10 725
District level healthcare	10 154	16 120
Primary level healthcare	9119	8096

OPD, outpatient department.

† , 01 January 2017 – 30 June 2017;

‡ , 01 January 2018 – 30 June 2018.

The EGK rejected an average of 95 tumour marker test requests per month from 01 January 2018 to 30 June 2018 with a total of 570 tests rejected over this period. Additionally, during the same 6-month period, no EGK-rejected tumour marker tests were restored. The total cost of tumour marker rejected test requests was R78 043.86 in 2018 (Table 3).

**TABLE 3:** Electronic gatekeeping rejection rate and costs saved in KwaZulu-Natal, South Africa, 01 January 2018 – 30 June 2018.

Tumour marker	Rejection rate due to EGK (%)	Cost of resulted samples 2018		Estimated cost of samples not run due to EGK	
		ZAR	USD	ZAR	USD
CA 125	09	9 903 797.00	54 137.27	913 572.00	499.39
PSA	14	2 146 656.00	117 342.98	3 088 008.00	1688.00
CA 19-9	09	8 122 332.00	44 399.23	710 556.00	388.41
CA 15-3	36	188 465.00	10 302.17	710 556.00	388.41
AFP	16	5 961 672.00	32 588.38	94 563.00	516.91
CEA	13	1 067 350.00	58 344.71	1 376 592.00	752.49
HCG	01	4 056 982.00	22 176.74	59 472.00	32.51

**Total**	**14**	**6 206 951.00**	**339 291.49**	**7 804 386.00**	**4266.12**

PSA, prostate-specific antigen; AFP, alpha foetal protein; CEA, carcinoembryonic antigen; CA 19-9, carbohydrate antigen 19-9; CA 125, cancer antigen 125; CA 15-3, cancer antigen 15-3; HCG, human chorionic gonadotropin; ZAR, South African rand (R); USD, United States dollar ($),

### Clinician questionnaire findings

We reviewed 22 responses from the 37 questionnaires distributed (59% response rate). Most respondents were from surgical departments (*n* = 24; 64%), followed by medical (*n* = 9; 24%), with the remainder (*n* = 3; 9%) being from general outpatient clinics or unspecified. Participants consisted predominantly of junior staff (interns) and non-specialist medical officers.

**FIGURE 3:**
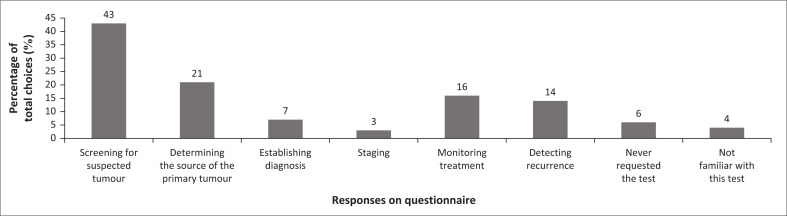
Reasons for requesting tumour marker testing per questionnaire respondents, June 2018, in KwaZulu-Natal, South Africa.

Ninety-five percent (*n* = 21) of respondents indicated that their facility had no dedicated oncology unit or clinics. A further 91% (*n* = 20) of the participants were unaware of any local or international tumour marker test request guidelines for clinical practice. Participants indicated the following as consequent actions to an abnormal tumour marker result: imaging studies (*n* = 20, 91%), 63% (*n* = 14) included biopsy, referral (*n* = 11, 50%), requesting another tumour marker test (*n* = 3, 14%), and 9% (*n* = 2) included repeating the tumour marker test. Most respondents requested tumour markers to query suspected tumours; over 20% (*n* = 4) indicated their use to detect tumour sources (Figure 3).

## Discussion

Tumour markers are some of the more expensive clinical chemistry tests. Based on the National Health Laboratory Service test pricing for the period 2017/2018, the total cost of tumour marker testing for the two periods reviewed was more than R10 million ($546 631.50 USD). All rejected tumour marker tests were estimated to cost R78 043.86 ($4266.12 USD) in 2018. In the public sector in South Africa, the cost of laboratory testing is paid by the Department of Health (state), with no cost to the patient. The EGK rejects test requests before payment and laboratory testing, hence no refund is made on rejected requests.

Previous reports from 1997–2012 state that 20% – 50% of laboratory tests are inappropriate or are not evidence-based practices.^22,23^ Pema, Kiabilua and Pillay (Gauteng, South Africa, in 2018),^15^ and Smit, Zemlin and Erasmus (Tygerberg, Western Cape, South Africa, in 2015)^14^ reported significant cost reductions through EGK of requests. However, the test requests reviewed were smaller-volume tests. Our findings showed that the number of tumour marker tests rejected by the EGK rules was minimal. Fewer than 20 tests were rejected on average, per month, for each of the tumour markers apart from PSA. This low rejection rate suggests that appropriate test ordering, per the test’s correct clinical requirement and guidelines, would be the most effective way of controlling inappropriate tumour marker test requests. Appropriate test ordering practice requires education on and routine reiteration of appropriate request guidelines, and the development and implementation of national testing guidelines. The lack of continuous clinician education is a reported driver of inappropriate testing.^24,25^ Education and continuous reiteration of best practices are especially important in the non-academic centres, where there are more generalists than specialists managing patients. The high request numbers from district health facilities support the fact that education regarding tumour marker utilisation is most needed in non-academic centres; however, district hospitals represent most hospitals servicing the population (*n* = 37) versus regional hospitals (*n* = 13).^26^ As evidenced by the respondents on the questionnaire, a lot of clinicians were requesting tumour markers to screen for malignancy.

Our findings may also be an indication that the EGK rules require further review and are not strict enough to achieve sufficient demand management. These rules could include limiting requests to only two tumour marker tests on one visit or admission to dissuade panel screening. However, stricter rules may not be possible for HCG, as it is also a test for normal pregnancy and pregnancy-related disorders (for example ectopic pregnancy) and serial measurements are critical. Human chorionic gonadotrophin was frequently requested for younger patients, probably because the laboratory information system could not distinguish between malignancy-related and pregnancy-related HCG testing. Additionally, germ cell tumours in which HCG concentrations may be increased are more frequently seen in younger adults and adolescents.

This is the first study, to the authors’ knowledge, that examines the use of tumour markers in sub-Saharan healthcare facilities. The findings of this study suggest that repeat testing represents a small fraction of the cost associated with tumour marker requests and that inappropriate requests (use of all tumour markers as screening tests) are likely resulting in test overuse and associated increased healthcare costs. The introduction of EGK has made little or no impact on the number and cost of tumour marker tests performed. While there are no national consensus guidelines for the utilisation of tumour markers in South Africa, international guidelines or best practice documents are available to guide clinicians to order tests appropriately.^27,28,29^

We recommend developing local and national tumour marker ordering guidelines for all levels of healthcare. Focused education at the undergraduate level and continuous professional development regarding appropriate utilisation of laboratory tests including tumour markers is also required. Greater involvement of pathologists in spreading appropriate utilisation awareness and coaching of junior doctors is also essential.

The increasing demands on limited healthcare resources and funding necessitate careful management of testing to ensure optimal patient care while managing costs.

### Limitations

One of the limitations of this study was that access to histology results was not available. Thus, tumour marker test results could not be confirmed by tumour biopsy results. Furthermore, the small number of questionnaires distributed may not be representative of the clinician cohort. Additionally, we did not sample clinicians from different healthcare facility levels. Furthermore, due to the geographical limitations, lack of internet availability, and other limited resources, we restricted questionnaire distribution to facilities within KwaZulu-Natal. In addition to that, HCG results were not separated into pregnant versus non-pregnant due to missing clinical records on many samples. Lastly, the effects of interventions to improve clinician knowledge of tumour marker requests were not assessed.

### Conclusion

It appears that many clinicians do not appropriately request and utilise tumour marker tests and there is no guideline for tumour test ordering and result interpretation. The EGK barely reduced tumour marker requests and costs as there was an increase in costs and testing numbers, despite EGK implementation. Education of doctors, stricter EGK rules, and additional demand management measures may be required to make a noticeable demand difference.
